# BUILDING BRIDGES TO CONDUCT RESEARCH WITH FAMILIES LIVING WITH COGNITIVE IMPAIRMENT IN THE US AND THE CARIBBEAN

**DOI:** 10.1093/geroni/igad104.1075

**Published:** 2023-12-21

**Authors:** Glenna Brewster

**Affiliations:** Emory University, Atlanta, Georgia, United States

## Abstract

My program of research aims to tailor and/or develop individual or dyadic behavioral and/or psychoeducational interventions to improve sleep and quality of life for persons living with cognitive impairment and their care partners. In order to conduct this research, it has been important to collaborate with colleagues in other disciplines and work beyond the borders of the United States. During this presentation, I will reflect on my research experience thus far using that to share practical strategies I used to develop and maintain collaborative relationships with researchers and stakeholders, and lessons learned from working within research teams locally, nationally and internationally.

